# Poly[[aqua­bis(μ_3_-isonicotinato-κ^3^ *O*:*O*′:*N*)tris­(μ_2_-isonicotinato-κ^3^ *O*,*O*′:*N*)(nitrato-κ*O*)bis­(μ_4_-oxalato-κ^6^ *O* ^1^,*O* ^2^:*O* ^2^:*O* ^1′^,*O* ^2′^:*O* ^1′^)dierbium(III)tetra­silver(I)] tetra­hydrate]

**DOI:** 10.1107/S160053680901842X

**Published:** 2009-05-23

**Authors:** Yan-Mei Wen, Tian-Jun Feng, Li-Xin He

**Affiliations:** a College of Science, Guangdong Ocean University, Zhanjiang 524088, People’s Republic of China; b College of Mathematics, Physics and Software Engineering, Lanzhou Jiaotong University, Lanzhou 730070, People’s Republic of China; c School of Pharmacy, Guangdong Phamaceutical University, Guangzhou 510006, People’s Republic of China

## Abstract

In the title coordination polymer, {[Ag_4_Er_2_(C_6_H_4_NO_2_)_5_(C_2_O_4_)_2_(NO_3_)(H_2_O)]·4H_2_O}_*n*_, each Er^III^ atom is coordinated in a bicapped trigonal–prismatic coordination geometry by three O atoms from two isonicotinate (IN) ligands, four O atoms from two oxalate ligands and one O atom from either a nitrate ion or a water mol­ecule, both of which are half-occupied over the same site. One Ag^I^ atom has a Y-shaped geometry defined by one N atom from one IN ligand, one O atom from another IN ligand and one O atom from an oxalate ligand. The other Ag^I^ atom is coordinated by two IN ligands and one O atom from an oxalate ligand. One of the IN ligands is disordered over an inversion center and forms a bridge between two centrosymmetric Ag^I^ ions. Due to the disorder, this IN ligand coordinates to the Ag atom through either the pyridyl N or the carboxyl­ate O atoms. The IN and oxalate ligands link the Er and Ag atoms into a three-dimensional coordination framework. O—H⋯O and C—H⋯O hydrogen bonds are observed in the crystal structure.

## Related literature

For general background to coordination polymers and open framework materials, see: Barbour (2006 ▶); Kepert (2006 ▶); Kong *et al.* (2008 ▶); Rao *et al.* (2004 ▶); Zhang *et al.* (2005 ▶). For background to isonicotinate complexes, see: Gheorghe *et al.* (2002 ▶).
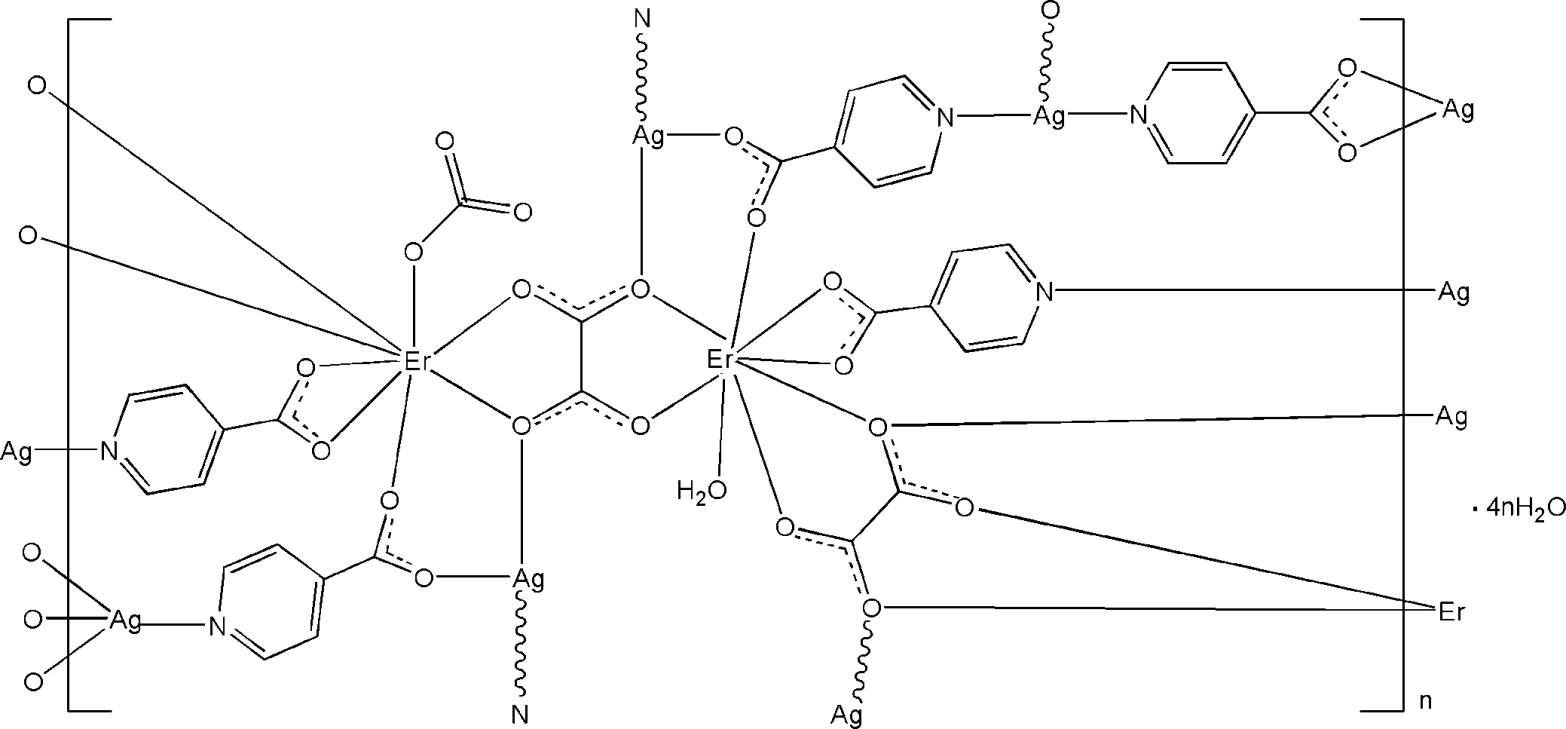


## Experimental

### 

#### Crystal data

[Ag_4_Er_2_(C_6_H_4_NO_2_)_5_(C_2_O_4_)_2_(NO_3_)(H_2_O)]·4H_2_O
*M* *_r_* = 1704.64
Triclinic, 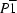

*a* = 8.8561 (8) Å
*b* = 11.6428 (8) Å
*c* = 11.9597 (9) Å
α = 76.940 (1)°
β = 76.612 (1)°
γ = 76.035 (1)°
*V* = 1145.14 (16) Å^3^
*Z* = 1
Mo *K*α radiation
μ = 5.40 mm^−1^
*T* = 296 K
0.30 × 0.25 × 0.21 mm

#### Data collection

Bruker APEXII CCD diffractometer
Absorption correction: multi-scan (*SADABS*; Sheldrick, 1996 ▶) *T*_min_ = 0.224, *T*_max_ = 0.336
5823 measured reflections
4043 independent reflections
3265 reflections with *I* > 2σ(*I*)
*R*_int_ = 0.028

#### Refinement

*R*[*F* ^2^ > 2σ(*F* ^2^)] = 0.053
*wR*(*F* ^2^) = 0.128
*S* = 1.06
4043 reflections
377 parameters
95 restraints
H-atom parameters constrained
Δρ_max_ = 1.57 e Å^−3^
Δρ_min_ = −1.32 e Å^−3^

### 

Data collection: *APEX2* (Bruker, 2007 ▶); cell refinement: *SAINT* (Bruker, 2007 ▶); data reduction: *SAINT*; program(s) used to solve structure: *SHELXS97* (Sheldrick, 2008 ▶); program(s) used to refine structure: *SHELXL97* (Sheldrick, 2008 ▶); molecular graphics: *SHELXTL* (Sheldrick, 2008 ▶) and *DIAMOND* (Brandenburg, 1999 ▶); software used to prepare material for publication: *SHELXTL*.

## Supplementary Material

Crystal structure: contains datablocks I, global. DOI: 10.1107/S160053680901842X/hy2195sup1.cif

Structure factors: contains datablocks I. DOI: 10.1107/S160053680901842X/hy2195Isup2.hkl

Additional supplementary materials:  crystallographic information; 3D view; checkCIF report

## Figures and Tables

**Table 1:** Selected bond lengths (Å)

Er1—O7	2.254 (7)
Er1—O11	2.339 (8)
Er1—O2^i^	2.364 (7)
Er1—O4^ii^	2.369 (7)
Er1—O3	2.371 (6)
Er1—O1	2.382 (6)
Er1—O5^iii^	2.400 (6)
Er1—O6^iii^	2.423 (7)
Ag1—N1	2.231 (8)
Ag1—O8	2.263 (7)
Ag1—O1	2.417 (7)
Ag2—N5	2.122 (9)
Ag2—N2	2.199 (8)
Ag2—O9^iv^	2.521 (9)
Ag2—O10^iv^	2.492 (9)

Symmetry codes: (i) 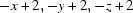; (ii) 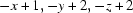; (iii) 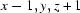; (iv) 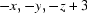.

**Table 2:** Hydrogen-bond geometry (Å, °)

*D*—H⋯*A*	*D*—H	H⋯*A*	*D*⋯*A*	*D*—H⋯*A*
O14—H14*A*⋯O9^v^	0.84	2.39	2.81 (3)	111
O14—H14*B*⋯O12^vi^	0.84	1.99	2.62 (3)	131
O16—H16*A*⋯O11^vii^	0.84	1.95	2.773 (12)	170
O16—H16*B*⋯O5^viii^	0.84	2.06	2.862 (12)	158
C6—H6⋯O2	0.93	2.53	3.371 (12)	151
C11—H11⋯O5^iii^	0.93	2.42	3.315 (8)	162
C12—H12⋯O14^ix^	0.93	2.56	3.441 (2)	158

Symmetry codes: (iii) 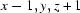; (v) 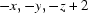; (vi) 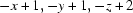; (vii) 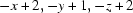; (viii) 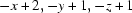; (ix) 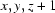.

**Fig. 1.**
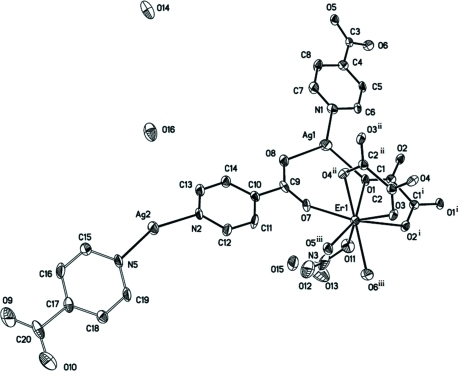
The asymmetric unit of the title compound, with the symmetry-related atoms to complete the Er coordination. Displacement ellipsoids are drawn at the 50% probability level. H atoms have been omitted for clarity. [Symmetry code: (i) 2 - x, 2 - y, 2 - z; (ii) 1 - x, 2 - y, 2 - z; (iii) -1 + x, y, 1 + z; (iv) -x, -y, 3 - z.]

**Fig. 2.**
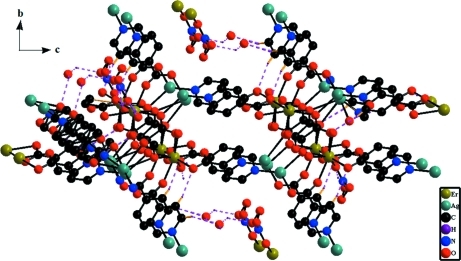
A packing view of the title compound. Hydrogen bonds are shown as dashed lines.

## supplementary crystallographic information

### Comment

The design and construction of transition-lanthanide metal complexes have gained great recognition over the last decade because of their intriguing network topologies and potential applications, and due to their magnetic properties, their capacity for gas storage, as luminescent materials, and so on (Barbour, 2006; Kepert, 2006; Kong *et al.*, 2008; Rao *et al.*, 2004; Zhang *et al.*, 2005). Isonicotinic acid is a multifunctional bridging ligand possessing of oxygen and nitrogen donors, which can thus be utilized to construct lanthanide-transition heterometallic complexes *via* the carboxyl oxygen atoms binding to lanthanides and nitrogen atoms bonding to transition metal ions such as Ag^I^ or Cu^I^ ions (Gheorghe *et al.*, 2002). On the basis of above considerations, we chose nicotinic acid, mixed 4 d–4f metal ions and nitric acid as our building blocks. A new three-dimensional 4 d–4f coordination framework resulted from the hydrothermal treatment of Er_2_O_3_, AgNO_3_, oxalic acid, isonicotinic acid and nitric acid in water.

As depicted in Fig. 1, the asymmetric unit of the title compound contains one Er^III^ atom, two Ag^I^ atoms, two and a half IN ligands, two half oxalate anions, each on an inversion center, a half-occupied nitrate ion, a half-occupied coordinated water molecule and two uncoordinated water molecules. The Er^III^ atom is eight-coordinated in a bicapped trigonal prism coordination geometry by three O atoms from two IN ligands, four O atoms from two oxalate ligands and one O atom from either a nitrate ion or a water molecule, both of which are half-occupied over the same site (Table 1). The Ag1 atom is located in a Y-shaped configuration, defined by one N atom from one IN ligand, one O atom from another IN ligand and one O atom from one oxalate ligand. One of the IN ligands is disordered on an inversion center halfway between two Ag2 atoms and forms a bridge between the two Ag2 atoms. Due to the inversion disorder of this IN ligand, Ag2 is thus coordinated either to two N atoms or to a pyridyl N and two carboxylate O atoms from two IN ligands. In the crystal structure, a zigzag Er–oxalate–Ag chain is formed *via* the oxalate ligand. The chains are linked by pillared IN ligands to form a two-dimensional layer. The layers are further interconnected by Ag_2_(IN)_3_ units to form a three-dimensional coordination framework. The supramolecular structure are further stabilized by O—H···O and C—H···O hydrogen bonds involving the carboxylate groups, uncoordinated water molecules and disordered nitrate ions (Table 2; Fig. 2).

### Experimental

A mixture of Er_2_O_3_ (0.192 g, 0.5 mmol), AgNO_3_ (0.169 g, 1 mmol), isonicotinic acid (0.123 g, 1 mmol), oxalic acid (0.09 g, 1 mmol), HNO_3_ (0.12 ml) and H_2_O (10 ml) was placed in a 23 ml Teflon-lined reactor, which was heated to 433 K for 3 d and then cooled to room temperature at a rate of 10 K h^-1^. The pale-purple crystals obtained were washed with water and dried in air (yield 46% based on Er).

### Refinement

C-bound H atoms were positioned geometrically and refined as riding atoms, with C—H = 0.93 Å and with *U*_iso_(H) = 1.2*U*_eq_(C). Water H atoms were tentatively located in difference Fourier maps and were refined with distance restraints of O—H = 0.84 (1) and H···H = 1.35 (1) Å and with *U*_iso_(H) = 1.5*U*_eq_(O). The hightest peak is located 0.95 Å from Ag1 and the deepest hole is located 1.02 Å from Er1.

### Crystal data

**Table:**

[Ag_4_Er_2_(C_6_H_4_NO_2_)_5_(C_2_O_4_)_2_(NO_3_)(H_2_O)]·4H_2_O	*Z* = 1
*M**_r_* = 1704.64	*F*(000) = 808
Triclinic, *P*1	*D*_x_ = 2.472 Mg m^−^^3^
Hall symbol: -P 1	Mo *K*α radiation, λ = 0.71073 Å
*a* = 8.8561 (8) Å	Cell parameters from 2895 reflections
*b* = 11.6428 (8) Å	θ = 2.4–27.9°
*c* = 11.9597 (9) Å	µ = 5.40 mm^−^^1^
α = 76.940 (1)°	*T* = 296 K
β = 76.612 (1)°	Block, colorless
γ = 76.035 (1)°	0.30 × 0.25 × 0.21 mm
*V* = 1145.14 (16) Å^3^	

### Data collection

**Table:**

Bruker APEXII CCD diffractometer	4043 independent reflections
Radiation source: fine-focus sealed tube	3265 reflections with *I* > 2σ(*I*)
graphite	*R*_int_ = 0.028
φ and ω scans	θ_max_ = 25.2°, θ_min_ = 1.8°
Absorption correction: multi-scan (*SADABS*; Sheldrick, 1996)	*h* = −10→10
*T*_min_ = 0.224, *T*_max_ = 0.336	*k* = −9→13
5823 measured reflections	*l* = −12→14

### Refinement

**Table:**

Refinement on *F*^2^	Primary atom site location: structure-invariant direct methods
Least-squares matrix: full	Secondary atom site location: difference Fourier map
*R*[*F*^2^ > 2σ(*F*^2^)] = 0.053	Hydrogen site location: inferred from neighbouring sites
*wR*(*F*^2^) = 0.128	H-atom parameters constrained
*S* = 1.06	*w* = 1/[σ^2^(*F*_o_^2^) + (0.0536*P*)^2^ + 8.9026*P*] where *P* = (*F*_o_^2^ + 2*F*_c_^2^)/3
4043 reflections	(Δ/σ)_max_ = 0.001
377 parameters	Δρ_max_ = 1.57 e Å^−^^3^
95 restraints	Δρ_min_ = −1.32 e Å^−^^3^

### Fractional atomic coordinates and isotropic or equivalent isotropic displacement parameters (Å^2^)

**Table:**

	*x*	*y*	*z*	*U*_iso_*/*U*_eq_	Occ. (<1)
Er1	0.75631 (5)	0.84475 (4)	1.13533 (4)	0.02560 (13)	
Ag1	1.03061 (11)	0.73575 (8)	0.83111 (8)	0.0482 (3)	
Ag2	0.34657 (11)	0.20986 (8)	1.22733 (8)	0.0471 (2)	
C1	1.0226 (11)	0.9620 (8)	0.9516 (8)	0.027 (2)	
C2	0.4797 (11)	1.0449 (8)	1.0425 (8)	0.027 (2)	
C3	1.5447 (11)	0.8125 (8)	0.3439 (9)	0.029 (2)	
C4	1.4291 (12)	0.7896 (9)	0.4583 (9)	0.034 (3)	
C5	1.4180 (12)	0.8533 (8)	0.5437 (9)	0.034 (3)	
H5	1.4845	0.9070	0.5338	0.041*	
C6	1.3046 (12)	0.8368 (8)	0.6469 (9)	0.035 (3)	
H6	1.2931	0.8834	0.7031	0.042*	
C7	1.2262 (14)	0.6927 (10)	0.5844 (10)	0.046 (3)	
H7	1.1616	0.6370	0.5969	0.056*	
C8	1.3334 (13)	0.7052 (10)	0.4786 (10)	0.044 (3)	
H8	1.3407	0.6585	0.4232	0.053*	
C9	0.8121 (10)	0.5945 (8)	1.0183 (9)	0.029 (2)	
C10	0.7093 (11)	0.5019 (8)	1.0682 (9)	0.030 (2)	
C11	0.5937 (12)	0.5111 (9)	1.1686 (10)	0.042 (3)	
H11	0.5839	0.5735	1.2082	0.050*	
C12	0.4945 (13)	0.4316 (9)	1.2108 (10)	0.040 (3)	
H12	0.4167	0.4421	1.2769	0.048*	
C13	0.6205 (13)	0.3255 (10)	1.0633 (10)	0.043 (3)	
H13	0.6291	0.2613	1.0264	0.052*	
C14	0.7232 (13)	0.4003 (9)	1.0175 (10)	0.039 (3)	
H14	0.8023	0.3851	0.9532	0.046*	
N1	1.2105 (10)	0.7535 (8)	0.6665 (8)	0.038 (2)	
N2	0.5076 (10)	0.3372 (7)	1.1577 (7)	0.032 (2)	
O1	0.9558 (8)	0.8752 (6)	0.9658 (6)	0.0322 (17)	
O2	1.1251 (8)	0.9947 (6)	0.8621 (6)	0.0335 (17)	
O3	0.5567 (8)	1.0219 (5)	1.1240 (6)	0.0308 (16)	
O4	0.3750 (8)	1.1387 (6)	1.0208 (6)	0.0323 (16)	
O5	1.5301 (8)	0.7718 (6)	0.2569 (6)	0.0358 (18)	
O6	1.6484 (8)	0.8716 (7)	0.3354 (6)	0.0425 (19)	
O7	0.8188 (8)	0.6624 (6)	1.0829 (6)	0.0359 (18)	
O8	0.8790 (8)	0.5966 (6)	0.9143 (6)	0.0387 (18)	
O11	0.9834 (9)	0.7560 (8)	1.2154 (8)	0.057 (2)	
H11A	1.0281	0.7809	1.2572	0.085*	0.50
H11B	0.9946	0.6776	1.2434	0.085*	0.50
N3	0.995 (4)	0.651 (2)	1.281 (2)	0.085 (8)	0.50
O12	0.860 (3)	0.612 (2)	1.318 (2)	0.088 (7)	0.50
O13	1.125 (3)	0.595 (3)	1.313 (3)	0.133 (12)	0.50
O14	0.209 (2)	0.3871 (14)	0.4571 (16)	0.063 (5)	0.50
H14A	0.1251	0.3651	0.4575	0.095*	0.50
H14B	0.1868	0.4304	0.5088	0.095*	0.50
O15	1.035 (3)	0.5205 (17)	1.2890 (18)	0.078 (6)	0.50
H15A	1.0173	0.4989	1.2315	0.117*	0.50
H15B	1.1234	0.4801	1.3018	0.117*	0.50
O16	0.7488 (11)	0.1435 (8)	0.8439 (9)	0.080 (3)	
H16A	0.8223	0.1814	0.8284	0.120*	
H16B	0.6798	0.1831	0.8045	0.120*	
N5	0.1892 (14)	0.1154 (11)	1.3561 (10)	0.037 (4)	0.50
C15	0.1322 (18)	0.0286 (12)	1.3241 (10)	0.042 (5)	0.50
H15	0.1631	0.0135	1.2482	0.051*	0.50
C16	0.029 (2)	−0.0355 (13)	1.4057 (13)	0.049 (5)	0.50
H16	−0.0092	−0.0935	1.3843	0.058*	0.50
C17	−0.0173 (19)	−0.0129 (14)	1.5192 (12)	0.035 (4)	0.50
C18	0.0398 (18)	0.0739 (14)	1.5512 (9)	0.039 (4)	0.50
H18	0.0088	0.0890	1.6271	0.047*	0.50
C19	0.1430 (16)	0.1380 (11)	1.4696 (11)	0.042 (5)	0.50
H19	0.1812	0.1960	1.4910	0.050*	0.50
C20	−0.136 (3)	−0.087 (2)	1.607 (3)	0.048 (5)	0.50
O9	−0.180 (3)	−0.162 (2)	1.5730 (17)	0.095 (7)	0.50
O10	−0.170 (2)	−0.0606 (18)	1.7064 (16)	0.070 (6)	0.50

### Atomic displacement parameters (Å^2^)

**Table:**

	*U*^11^	*U*^22^	*U*^33^	*U*^12^	*U*^13^	*U*^23^
Er1	0.0229 (2)	0.0283 (2)	0.0268 (2)	−0.01183 (17)	0.00013 (17)	−0.00509 (17)
Ag1	0.0470 (5)	0.0600 (6)	0.0354 (5)	−0.0242 (4)	0.0112 (4)	−0.0090 (4)
Ag2	0.0507 (5)	0.0476 (5)	0.0445 (5)	−0.0299 (4)	0.0100 (4)	−0.0089 (4)
C1	0.023 (5)	0.032 (5)	0.026 (5)	−0.009 (4)	0.002 (4)	−0.005 (4)
C2	0.026 (5)	0.027 (5)	0.027 (5)	−0.008 (4)	0.001 (4)	−0.004 (4)
C3	0.031 (5)	0.021 (5)	0.033 (6)	−0.006 (4)	−0.005 (4)	−0.003 (4)
C4	0.029 (5)	0.036 (6)	0.032 (6)	−0.004 (4)	0.002 (4)	−0.007 (5)
C5	0.043 (6)	0.023 (5)	0.035 (6)	−0.019 (4)	0.007 (5)	−0.002 (4)
C6	0.046 (6)	0.021 (5)	0.038 (6)	−0.010 (4)	−0.006 (5)	−0.004 (4)
C7	0.053 (7)	0.046 (6)	0.040 (7)	−0.032 (5)	0.014 (5)	−0.010 (5)
C8	0.049 (6)	0.042 (6)	0.044 (7)	−0.027 (5)	0.015 (5)	−0.019 (5)
C9	0.016 (4)	0.026 (5)	0.043 (6)	−0.009 (4)	−0.014 (4)	0.012 (4)
C10	0.030 (5)	0.028 (5)	0.035 (6)	−0.013 (4)	−0.012 (4)	0.003 (4)
C11	0.043 (6)	0.031 (5)	0.056 (7)	−0.021 (5)	0.013 (5)	−0.027 (5)
C12	0.047 (6)	0.037 (6)	0.040 (6)	−0.022 (5)	0.005 (5)	−0.015 (5)
C13	0.055 (7)	0.035 (6)	0.042 (7)	−0.026 (5)	0.002 (6)	−0.007 (5)
C14	0.042 (6)	0.035 (6)	0.038 (6)	−0.017 (5)	0.006 (5)	−0.010 (5)
N1	0.036 (5)	0.043 (5)	0.031 (5)	−0.016 (4)	0.009 (4)	−0.004 (4)
N2	0.042 (5)	0.021 (4)	0.036 (5)	−0.016 (4)	−0.002 (4)	−0.001 (3)
O1	0.034 (4)	0.025 (3)	0.036 (4)	−0.018 (3)	0.008 (3)	−0.005 (3)
O2	0.037 (4)	0.036 (4)	0.034 (4)	−0.023 (3)	0.003 (3)	−0.013 (3)
O3	0.033 (4)	0.024 (3)	0.039 (4)	−0.002 (3)	−0.011 (3)	−0.012 (3)
O4	0.031 (4)	0.037 (4)	0.032 (4)	−0.001 (3)	−0.009 (3)	−0.016 (3)
O5	0.036 (4)	0.032 (4)	0.039 (4)	−0.017 (3)	0.001 (3)	−0.004 (3)
O6	0.039 (4)	0.058 (5)	0.036 (4)	−0.034 (4)	0.006 (3)	−0.006 (3)
O7	0.038 (4)	0.022 (3)	0.049 (5)	−0.014 (3)	−0.005 (3)	−0.004 (3)
O8	0.041 (4)	0.035 (4)	0.042 (5)	−0.020 (3)	−0.001 (4)	−0.004 (3)
O11	0.052 (5)	0.055 (5)	0.068 (6)	−0.011 (4)	−0.033 (4)	0.001 (4)
N3	0.12 (2)	0.081 (17)	0.087 (18)	−0.060 (16)	−0.025 (17)	−0.036 (14)
O12	0.099 (15)	0.086 (14)	0.102 (17)	−0.043 (13)	−0.028 (13)	−0.030 (12)
O13	0.099 (19)	0.12 (2)	0.14 (2)	−0.004 (17)	−0.052 (18)	0.058 (18)
O14	0.054 (10)	0.040 (9)	0.082 (13)	−0.013 (8)	−0.005 (10)	0.015 (9)
O15	0.121 (17)	0.054 (11)	0.080 (14)	−0.036 (12)	−0.042 (13)	−0.010 (10)
O16	0.068 (6)	0.064 (6)	0.117 (9)	−0.029 (5)	−0.044 (6)	0.010 (6)
N5	0.041 (10)	0.028 (9)	0.043 (8)	−0.021 (7)	0.005 (8)	−0.003 (7)
C15	0.053 (12)	0.037 (11)	0.041 (10)	−0.017 (9)	−0.015 (8)	0.000 (8)
C16	0.055 (12)	0.039 (11)	0.062 (9)	−0.029 (9)	−0.007 (9)	−0.014 (8)
C17	0.020 (8)	0.029 (9)	0.057 (8)	−0.009 (6)	−0.009 (7)	−0.003 (7)
C18	0.032 (10)	0.043 (10)	0.042 (9)	−0.019 (7)	0.003 (8)	−0.007 (7)
C19	0.049 (12)	0.029 (10)	0.049 (10)	−0.025 (8)	0.002 (10)	−0.007 (8)
C20	0.026 (10)	0.029 (10)	0.075 (10)	−0.006 (7)	−0.011 (9)	0.018 (8)
O9	0.090 (13)	0.125 (15)	0.088 (14)	−0.089 (12)	0.001 (13)	0.001 (11)
O10	0.053 (11)	0.068 (13)	0.072 (10)	−0.016 (10)	0.005 (10)	0.011 (9)

### Geometric parameters (Å, °)

**Table:**

Er1—O7	2.254 (7)	C10—C11	1.392 (14)
Er1—O11	2.339 (8)	C10—C14	1.415 (15)
Er1—O2^i^	2.364 (7)	C11—C12	1.365 (14)
Er1—O4^ii^	2.369 (7)	C11—H11	0.9300
Er1—O3	2.371 (6)	C12—N2	1.357 (13)
Er1—O1	2.382 (6)	C12—H12	0.9300
Er1—O5^iii^	2.400 (6)	C13—N2	1.332 (13)
Er1—O6^iii^	2.423 (7)	C13—C14	1.350 (14)
Ag1—N1	2.231 (8)	C13—H13	0.9300
Ag1—O8	2.263 (7)	C14—H14	0.9300
Ag1—O1	2.417 (7)	O2—Er1^i^	2.364 (7)
Ag2—N5	2.122 (9)	O4—Er1^ii^	2.369 (7)
Ag2—N2	2.199 (8)	O5—Er1^v^	2.400 (6)
Ag2—O9^iv^	2.521 (9)	O6—Er1^v^	2.423 (7)
Ag2—O10^iv^	2.492 (9)	O11—N3	1.28 (3)
C1—O1	1.251 (11)	O11—H11A	0.8385
C1—O2	1.283 (11)	O11—H11B	0.8880
C1—C1^i^	1.536 (19)	N3—O13	1.28 (4)
C2—O3	1.260 (12)	N3—O12	1.32 (3)
C2—O4	1.270 (11)	O14—H14A	0.8385
C2—C2^ii^	1.545 (19)	O14—H14B	0.8436
C3—O6	1.248 (12)	O15—H15A	0.8402
C3—O5	1.280 (12)	O15—H15B	0.8410
C3—C4	1.519 (13)	O16—H16A	0.8365
C3—Er1^v^	2.762 (10)	O16—H16B	0.8405
C4—C5	1.365 (15)	N5—C15	1.3900
C4—C8	1.394 (14)	N5—C19	1.3900
C5—C6	1.407 (14)	C15—C16	1.3900
C5—H5	0.9300	C15—H15	0.9300
C6—N1	1.374 (13)	C16—C17	1.3900
C6—H6	0.9300	C16—H16	0.9300
C7—N1	1.299 (14)	C17—C18	1.3900
C7—C8	1.396 (15)	C17—C20	1.57 (3)
C7—H7	0.9300	C18—C19	1.3900
C8—H8	0.9300	C18—H18	0.9300
C9—O7	1.243 (12)	C19—H19	0.9300
C9—O8	1.246 (12)	C20—O9	1.22 (3)
C9—C10	1.509 (13)	C20—O10	1.24 (3)
			
O7—Er1—O11	78.4 (3)	C14—C10—C9	123.0 (9)
O7—Er1—O2^i^	140.7 (2)	C12—C11—C10	122.1 (10)
O11—Er1—O2^i^	74.3 (3)	C12—C11—H11	118.9
O7—Er1—O4^ii^	73.3 (2)	C10—C11—H11	118.9
O11—Er1—O4^ii^	146.0 (3)	N2—C12—C11	121.0 (10)
O2^i^—Er1—O4^ii^	118.2 (2)	N2—C12—H12	119.5
O7—Er1—O3	139.7 (2)	C11—C12—H12	119.5
O11—Er1—O3	141.7 (3)	N2—C13—C14	124.5 (10)
O2^i^—Er1—O3	73.1 (2)	N2—C13—H13	117.7
O4^ii^—Er1—O3	69.5 (2)	C14—C13—H13	117.7
O7—Er1—O1	78.8 (2)	C13—C14—C10	119.4 (10)
O11—Er1—O1	79.9 (3)	C13—C14—H14	120.3
O2^i^—Er1—O1	69.1 (2)	C10—C14—H14	120.3
O4^ii^—Er1—O1	76.4 (2)	C7—N1—C6	117.5 (9)
O3—Er1—O1	106.1 (2)	C7—N1—Ag1	123.2 (7)
O7—Er1—O5^iii^	83.7 (2)	C6—N1—Ag1	119.2 (7)
O11—Er1—O5^iii^	110.4 (3)	C13—N2—C12	117.6 (9)
O2^i^—Er1—O5^iii^	132.3 (2)	C13—N2—Ag2	122.1 (7)
O4^ii^—Er1—O5^iii^	85.0 (2)	C12—N2—Ag2	120.4 (7)
O3—Er1—O5^iii^	78.5 (2)	C1—O1—Er1	118.2 (6)
O1—Er1—O5^iii^	157.4 (2)	C1—O1—Ag1	121.9 (6)
O7—Er1—O6^iii^	122.2 (2)	Er1—O1—Ag1	119.9 (3)
O11—Er1—O6^iii^	81.4 (3)	C1—O2—Er1^i^	118.2 (6)
O2^i^—Er1—O6^iii^	81.1 (2)	C2—O3—Er1	117.8 (6)
O4^ii^—Er1—O6^iii^	130.0 (2)	C2—O4—Er1^ii^	118.2 (6)
O3—Er1—O6^iii^	74.3 (2)	C3—O5—Er1^v^	92.1 (6)
O1—Er1—O6^iii^	148.1 (2)	C3—O6—Er1^v^	91.9 (6)
O5^iii^—Er1—O6^iii^	54.4 (2)	C9—O7—Er1	150.9 (6)
N1—Ag1—O8	132.0 (3)	C9—O8—Ag1	118.1 (6)
N1—Ag1—O1	124.5 (3)	N3—O11—Er1	120.6 (14)
O8—Ag1—O1	103.6 (2)	N3—O11—H11A	91.9
N5—Ag2—N2	156.4 (4)	Er1—O11—H11A	130.9
O1—C1—O2	125.7 (9)	Er1—O11—H11B	115.6
O1—C1—C1^i^	117.7 (10)	H11A—O11—H11B	103.8
O2—C1—C1^i^	116.6 (10)	O13—N3—O11	121 (3)
O3—C2—O4	125.6 (9)	O13—N3—O12	123 (3)
O3—C2—C2^ii^	117.8 (10)	O11—N3—O12	115 (3)
O4—C2—C2^ii^	116.5 (11)	O13—N3—H11B	120.8
O6—C3—O5	121.5 (9)	O12—N3—H11B	110.8
O6—C3—C4	120.3 (9)	N3—O13—H15B	100.2
O5—C3—C4	118.2 (9)	H14A—O14—H14B	106.6
O6—C3—Er1^v^	61.3 (5)	H15A—O15—H15B	106.7
O5—C3—Er1^v^	60.3 (5)	H16A—O16—H16B	107.4
C4—C3—Er1^v^	177.8 (7)	C15—N5—C19	120.0
C5—C4—C8	118.8 (9)	C15—N5—Ag2	118.0 (7)
C5—C4—C3	119.4 (9)	C19—N5—Ag2	122.0 (7)
C8—C4—C3	121.8 (10)	N5—C15—C16	120.0
C4—C5—C6	119.2 (10)	N5—C15—H15	120.0
C4—C5—H5	120.4	C16—C15—H15	120.0
C6—C5—H5	120.4	C15—C16—C17	120.0
N1—C6—C5	121.6 (10)	C15—C16—H16	120.0
N1—C6—H6	119.2	C17—C16—H16	120.0
C5—C6—H6	119.2	C18—C17—C16	120.0
N1—C7—C8	124.5 (10)	C18—C17—C20	122.1 (15)
N1—C7—H7	117.8	C16—C17—C20	117.9 (15)
C8—C7—H7	117.8	C17—C18—C19	120.0
C4—C8—C7	118.2 (10)	C17—C18—H18	120.0
C4—C8—H8	120.9	C19—C18—H18	120.0
C7—C8—H8	120.9	C18—C19—N5	120.0
O7—C9—O8	126.5 (9)	C18—C19—H19	120.0
O7—C9—C10	117.7 (9)	N5—C19—H19	120.0
O8—C9—C10	115.7 (9)	O9—C20—O10	128 (2)
C11—C10—C14	115.3 (9)	O9—C20—C17	118 (2)
C11—C10—C9	121.7 (9)	O10—C20—C17	113 (2)

Symmetry codes: (i) −*x*+2, −*y*+2, −*z*+2; (ii) −*x*+1, −*y*+2, −*z*+2; (iii) *x*−1, *y*, *z*+1; (iv) −*x*, −*y*, −*z*+3; (v) *x*+1, *y*, *z*−1.

### Hydrogen-bond geometry (Å, °)

**Table:**

*D*—H···*A*	*D*—H	H···*A*	*D*···*A*	*D*—H···*A*
O14—H14A···O9^vi^	0.84	2.39	2.81 (3)	111
O14—H14B···O12^vii^	0.84	1.99	2.62 (3)	131
O16—H16A···O11^viii^	0.84	1.95	2.773 (12)	170
O16—H16B···O5^ix^	0.84	2.06	2.862 (12)	158
C6—H6···O2	0.93	2.53	3.371 (12)	151
C11—H11···O5^iii^	0.93	2.42	3.315 (8)	162
C12—H12···O14^x^	0.93	2.56	3.441 (2)	158

Symmetry codes: (vi) −*x*, −*y*, −*z*+2; (vii) −*x*+1, −*y*+1, −*z*+2; (viii) −*x*+2, −*y*+1, −*z*+2; (ix) −*x*+2, −*y*+1, −*z*+1;  (iii) *x*−1, *y*, *z*+1; (x) *x*, *y*, *z*+1.
